# SAW-Based Phononic Crystal Microfluidic Sensor—Microscale Realization of Velocimetry Approaches for Integrated Analytical Platform Applications

**DOI:** 10.3390/s17102187

**Published:** 2017-09-23

**Authors:** Aleksandr Oseev, Ralf Lucklum, Mikhail Zubtsov, Marc-Peter Schmidt, Nikolay V. Mukhin, Soeren Hirsch

**Affiliations:** 1 Institute of Micro and Sensor Systems (IMOS), Otto-von-Guericke-University, 39106 Magdeburg, Germany; ralf.lucklum@ovgu.de (R.L.); mikhail.zubtsov@ovgu.de (M.Z.); marc-peter.schmidt@ovgu.de (M.-P.S.); mukhin.nikolay.v@gmail.com (N.V.M.); 2 Department of Electronics, Saint Petersburg Electrotechnical University “LETI”, Saint Petersburg 197376, Russia; 3 Department of Engineering, University of Applied Sciences Brandenburg, 14770 Brandenburg, Germany; soeren.hirsch@fh-brandenburg.de

**Keywords:** phononic crystal, liquid sensor, microfluidic sensor, ultrasonic velocimetry, μTAS

## Abstract

The current work demonstrates a novel surface acoustic wave (SAW) based phononic crystal sensor approach that allows the integration of a velocimetry-based sensor concept into single chip integrated solutions, such as Lab-on-a-Chip devices. The introduced sensor platform merges advantages of ultrasonic velocimetry analytic systems and a microacoustic sensor approach. It is based on the analysis of structural resonances in a periodic composite arrangement of microfluidic channels confined within a liquid analyte. Completed theoretical and experimental investigations show the ability to utilize periodic structure localized modes for the detection of volumetric properties of liquids and prove the efficacy of the proposed sensor concept.

## 1. Introduction

The integration of analytical instruments into miniaturized platforms has gained a significant interest for a broad variety of applications. Single chip integrated solutions, such as Lab-on-a-Chip and Micro Total Analysis Systems (μTAS), require the use of compatible approaches to perform the functional elements of analytical systems. These systems allow one to analyze complex liquid probes non-invasively within a single analytical chip, and obtain all necessary information from sample volumes up to the picoliter scale [1,2,3,4].

The acoustic methods of liquid analysis are widely utilized nowadays for chemical sensors, biosensors, and in medical applications [5]. The information on an analyzed solution can be obtained from: volumetric properties analysis, such as the velocity of sound, density, and viscosity; attenuation of the wave in liquid medium; mass load of absorbed analyte on the propagation path; and many other approaches. In the micrometer wavelength range, microacoustic devices provide a well-established platform for sensor realization [6]. The application of surface acoustic wave (SAW) devices for sensing purposes is rather specific depending on the medium that is analyzed. Since the detection with SAW sensors is realized as a result of the interrogation of the surface wave with the detecting medium, a higher acoustic energy density at the waveguide/liquid boundary is preferred. Most of the broadly utilized SAW sensor concepts are based on the surface interrogation of the acoustic waves that are localized at the piezoelectric boundary and the material at the interface [7]. The higher the acoustic energy surface localization, the higher the sensitivity that can presumably be reached. In terms of liquid sensor applications, it is necessary to utilize specific types of surface waves that avoid the radiation of acoustic energy into the liquid volume (in the case of Rayleigh waves, for instance), which leads to a significant attenuation of the propagating wave. For that reason, the special types of piezoelectric substrates that support the propagation of horizontally polarized wave types or the structures with the excitation of low-speed sound acoustic plate modes should be used. The basic concept of most of the developed SAW-based liquid sensors is focused on the detection of the mass load of evanescent moved liquid within the penetration depth that can be correlated with the analyte mass density and viscosity [8]. 

Flexural plate mode (FPW) sensors were applied for the detection of the liquid properties. Based on the propagation of Lamb waves through the membranes, they exhibit high sensitivity to mass load. Due to low Lamb wave acoustic velocity, the radiation of acoustic energy into the liquid volume is reduced and limited in most cases only by an evanescent acoustic disturbance [9,10]. Horizontally polarized shear wave devices on a basis of lithium niobate and lithium tantalite substrates of different cuts allowed the sensor to operate with high permittivity liquids, even in cases where it is directly applied to the waveguide surface. Thus, the SAW sensor based on a 36° YX cut of LiTaO_3_ wafer was utilized as a biosensor to detect amounts of an enzyme immobilized on a surface during the catalytic reaction [11]. Another type of shear horizontal SAW liquid sensor was developed with the use of Love surface waves. This type of wave is localized within the over-layer with low acoustic wave velocity. Such acoustic wave localization allows for the reduction of the surface wave attenuation caused by scattering into the bulk of the substrate. The adjustment of the waveguide layer thickness may create an acoustic film resonance that significantly improves the sensor sensitivity to mass loading [12].

The SAW-based sensor concepts were broadly utilized for biosensor applications. In most cases, the sensor structure is formed on a solid substrate basis with a tailored wave propagation path (surface modification with recognition layers), which allow for the realization of liquid sensors that are specifically sensitive to certain targeting substances. The detecting variation of mass load in this case is extended by altering the functional layer properties caused by the adsorption in a sensitive layer or binding to the recognition layer. In contrast to near-surface detection mechanisms, the velocimetry-based sensor approaches detect the variation of the speed of sound of the liquid analyte. Excitation of liquid pressure resonances and control of the resonant response of liquid-containing volumes is one of the most convenient ways to measure the analyte velocity of sound. The application of cavity-based approaches on a basis of SAW sensor platform is rather challenging because of the unavoidable scattering of acoustic waves into the substrate volume. Therefore, the velocimetry-based analysis of liquids on a SAW sensor platform has certain limitations. In our previous work, we have already demonstrated the advantages of the phononic crystal liquid sensor concept that allows the detection of the speed of sound of liquids. In this current contribution, we show one of the approaches to integrate the same concept into a planar SAW-based sensor platform.

Methods of ultrasonic velocimetry that have a development background similar to microacoustic devices are able to conduct the volumetric properties analysis of liquid analytes [13]. The significant advantage of the method is an ability to gain information from the intermolecular interactions of liquid mixtures. The reaction on molecule interactions is reflected in liquid compressibility that can be detected by probing the analyte with ultrasonic waves. Measurements of sound velocity at a certain range of pressures can evaluate thermodynamic quantities of the liquid analyte. As a result, the intermolecular forces that can be estimated from the compressibility data determine the thermodynamic parameters of the analyzed solution. In recent years, the methods of high resolution ultrasonic spectroscopy have achieved significant improvements in resolution and have become widely used for sensing purposes. The ultrasonic velocimetry approach is a powerful tool that has a broad variety of applications. However, nowadays demands require further sensor miniaturization and the ability to be integrated into a multifunctional platform on a single chip. That makes it necessary to develop novel sensor platforms that meet such requirements and, at the same time, maintain all the advantages of the already developed concept.

The developments of micromachined ultrasonic transducers introduced the platform that has been successfully utilized for biomedical imaging applications in the last decade [14]. Immersion types of capacitive transducers (CMUT) were utilized for ultrasonic diagnostic processes to screen biomedical substances and deduce information from the reflected ultrasound [15]. The transducers can be batch-fabricated with high quality and uniformity across the array. CMUT arrays were successfully applied for the imaging of biological objects at certain area in a broad frequency range performing pulse-echo measurements or other measurement approaches [16]. The broadband operation frequency range, which is advantageous for the imaging applications, has a consequence of relatively high insertion losses that, in comparison with resonant microacoustic sensor approaches, appear as a shortcoming of the CMUT platform for sensing applications.

The recently introduced phononic crystal (PnC) sensor platform [17,18,19] provides an alternative solution for the sensor realization that utilizes the advantages of ultrasonic velocimetry methods and can be built on the basis of the developed microacoustic sensor platform [20], fulfilling the required integration to planar Lab-on-a-Chip solutions. The most critical point is the ability of the novel sensor system to satisfy high demands regarding the sensitivity that the developed sensor platforms have already demonstrated. The ability to measure a variation in the speed of sound up to 0.1 m/s is the most critical point regarding the sensor performance. The current development state of phononic crystal liquid sensors demonstrates the ability of the concept to satisfy the sensor demands. In order to make the approach competitive to existing sensor systems and satisfy the Lab-on-a-Chip integration requirements, the respective sensor design and fabrication technology have to be developed. In this current article, we demonstrate a SAW-based phononic crystal sensor structure that is fabricated as a periodically distributed arrangement of microfluidic channels. We demonstrate the structure design, the fabrication technology, and the results of the sensor. Moreover, experimental verification proves the feasibility of the proposed SAW-based sensor concept and provides a sufficient basis for further sensor development. Taking the advantages of intermolecular interaction sensing, the developed and described sensor platform can be utilized for the detection of binding processes of proteins, antigen—antibody interactions, the binding of cations by polynucleotides, and many others molecular interactions. The influence of solute-solvent interactions on mixtures’ velocity of sound opens the possibility of analyzing various biological analytes. Therefore, the developed sensor platform can be utilized for various biomedical applications similar to existing methods, such as immunoenzyme analysis [21] or the control of blood clotting [22]. Moreover, it can assist in medical applications via analyses for the detection of cancer cells [23], the diagnosis of gastritis [24], and many other applications.

## 2. Materials and Methods 

**Figure 1:**
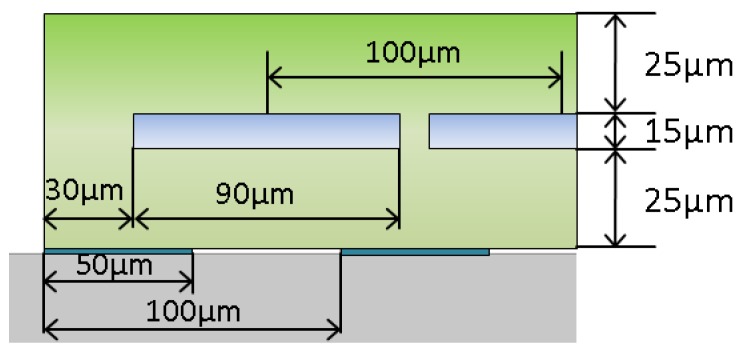
Dimensions of the microfluidic structure and the SAW electrodes geometry.

Lithium niobate (LiNbO_3_) 127.86° Y-cut with wave propagation in the X direction was chosen as a substrate material. It has an efficient coupling to Rayleigh wave and is a typical material for broadband SAW devices. Electrodes with an equal aperture of 100 of SAW wavelengths (unapodized interdigitated transducers IDTs), an equal electrodes width, and a period along the wave propagation (equidistant IDTs) were used in the current sensor design. The electrodes and contact bars were arranged as a two port SAW device. No passive electrodes or reflective gratings were applied. The microfluidic sensor arrangement was fabricated on top of the receiving IDTs. The fluidic part of the sensor is defined as the part between the interfacial and covering polymer layers. The major role of the interfacial layer is to couple an incident surface acoustic wave to the microfluidic structure. The channels are closed from above with another polymer layer to encapsulate the liquid-containing volumes. The interfacial layer was found to be optimal with a thickness equal to one quarter of the wavelength. SU-8 polymer was chosen as the construction material for the interfacial layer. The material parameters used in numerical model were defined as follows: Young’s modulus of 4.95 GPa and density 1200 kg/m^3^. The thickness of the layer was estimated as 25 μm for the central frequency of 20 MHz. From the symmetry reasons, the covering SU-8 layer was completed with the same thickness of 25 μm. The height of the microfluidic channels was found to be optimal when equal to 1/5 of the wavelength in liquid domains (for the speed of sound of 1500 m/s). The relation of channel width and channel period was defined as a result of completed simulations. The influence of the number of channels and the channel filling factor were previously theoretically investigated [25]. The device described in the current work was a periodic arrangement forming a system of four microfluidic channels. The complete structure dimensions are shown in Figure 1. 

The following materials were utilized for the fabrication processes of the sensor structure. Titanium and aluminum targets were used in the physical vapor deposition (PVD) sputtering process and were supplied by a local manufacturer. The structuring of metal layers was completed by applying TI35ES-positive photoresist together with TI Prime adhesive promoter, which were supplied by MicroChemicals GmbH (Ulm, Germany). AZ Dev photoresist developer as well as acetone, isopropanol, ANPE aluminum etcher, and other supplies were obtained from MicroChemicals GmbH as well. The structuring of SU-8 50 and SU-8 5 microfluidic channels was completed with mr600Dev developer supplied by Micro Resist Technology GmbH (Berlin, Germany). 

The deposition of the metal layers was completed with LS 500 ES physical vapor deposition equipment. Spin-coating processes were completed with SUSS Labspin manufacturing line. The lithography steps were made with SUSS MA6/BA6 mask aligner. The processes of SU-8 adhesive bonding were conducted in an SUSS SB6E substrate bonder. The manufactured SAW-based phononic crystal sensor was analyzed with a ZEISS EVO 50 scanning electron microscope and FTR MicroProf 300 profilometer.

**Figure 2:**
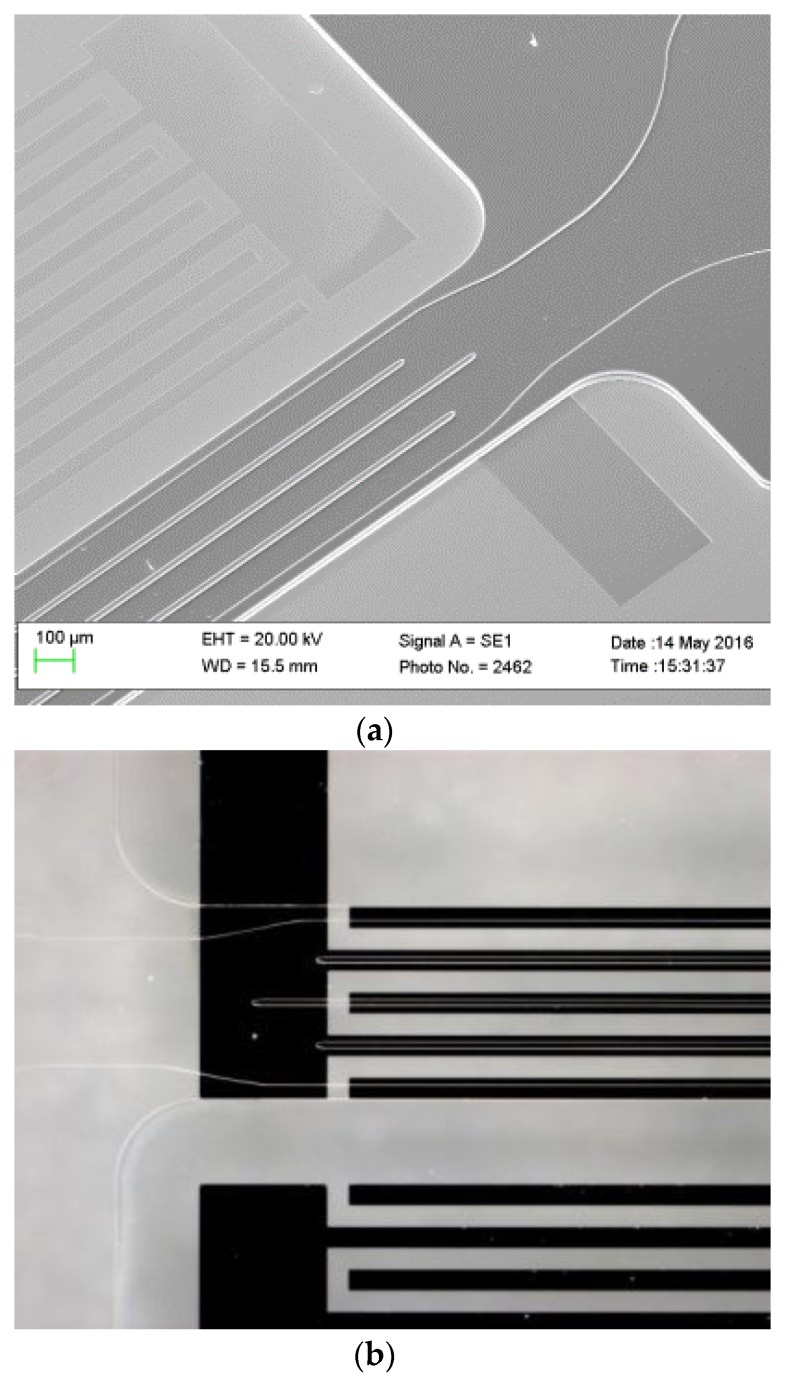
(**a**) SEM images of the fabricated microfluidic structures atop the interfacial layer; (**b**) microscope image of the completed SAW-based phononic microfluidic structure.

The fabrication process was separated into several parts such as the manufacture of IDTs, the fabrication of the opened periodic microfluidic structure, and the channels encapsulation. The aluminum IDTs were fabricated prior to the microfluidic structure processing. The performance of the microfluidic arrangement plays a significant role in the sensor response that leads to restricted fabrication tolerances. As a basis for the microfluidic arrangement realization, SU-8 polymer-based technology was applied. Chemically stable against most solvents, the epoxy-based SU-8 photoresist was previously recognized as a promising solution for building microfluidic structures. Despite the advantages of this polymer, its usage is associated with the adjustment of up to 30 fabrication parameters of the respective technological process in order to achieve designed structures. The utilization of the SU-8 adhesive bonding is an advantage of that polymer, but in order to achieve a sufficient bonding quality, the manufacturing process should be optimized for a certain structure design. A scanning electron microscope (SEM) image of the fabricated structure is shown in Figure 2a. The SEM image was taken from the opened structure that was subsequently encapsulated with a covering polymer layer. The microscope image of the completed structure is illustrated in Figure 2b. Because SU-8 polymer is optically transparent, only the structure boundaries can be distinctively observed.

## 3. Results

**Figure 3:**
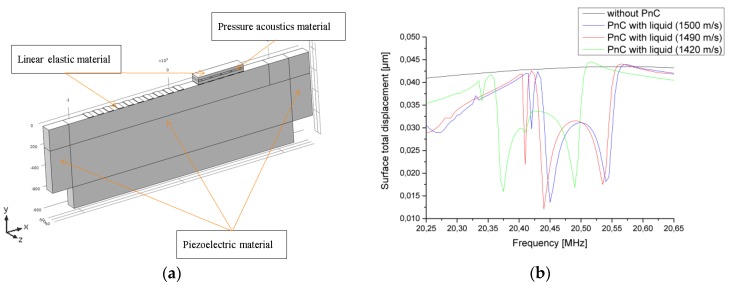
(**a**) Computation model with specified domains; (**b**) Computed structure transmission response obtained as a total displacement taken from the receiving IDTs boundaries for the speed of sound of channels confined liquid of 1500 m/s (orange curve), 1490 m/s (blue curve), 1420 m/s (pink curve), and SAW transmission response without the microfluidic structure (black curve).

The computation model is demonstrated in Figure 3a. The simulations were completed with an acoustic module of COMSOL^TM^ Multiphysics software (Burlington, MA, USA). The structure geometry was divided into separate domains of three different types, each of which is described with a separate system of equations. The “piezoelectric material” domains (waveguide and perfectly matched layer PML domains) are described as anisotropic piezoelectric materials. The 127.86° Y cut is defined with a rotational coordinate system that recalculates the respective material properties in accordance with Euler angles prescribed in the rotation coordinate system of the model. The computational domains of the polymer microfluidic structures and IDTs are described as isotropic materials that are mechanically coupled to the piezoelectric waveguide. These domains are described as “linear elastic material” domains. The liquid that fills the microfluidic channels is described by the pressure acoustic model. It specifies the propagation of pressure waves in the liquid domains and contains the liquid material properties that are targeted for sensing (such as speed of sound and density). Model boundaries in the Z planes are prescribed with periodic boundary conditions that make the whole arrangement infinitely long in the Z direction. This boundary condition allows for a significant reduction of the computational model to complete the simulation tasks within a meaningful time duration. The surface acoustic wave excitation is completed with prescribed periodic potential and ground boundaries along the X direction of the waveguide surface. Receiving IDTs-waveguide boundaries are prescribed with “ground” and “float potential” conditions. The receiving electrodes periodicity is also wavelength fold. The microfluidic structure is placed directly above the receiving IDTs. The frequency region within which the simulation model response demonstrates the most of sensitivity depending on the speed of sound of the liquid domains is displayed in Figure 3b.

**Figure 4:**
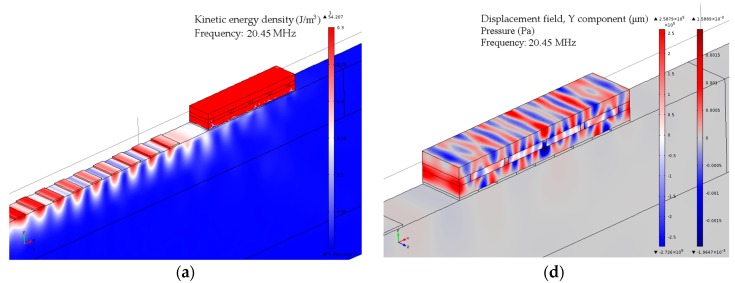

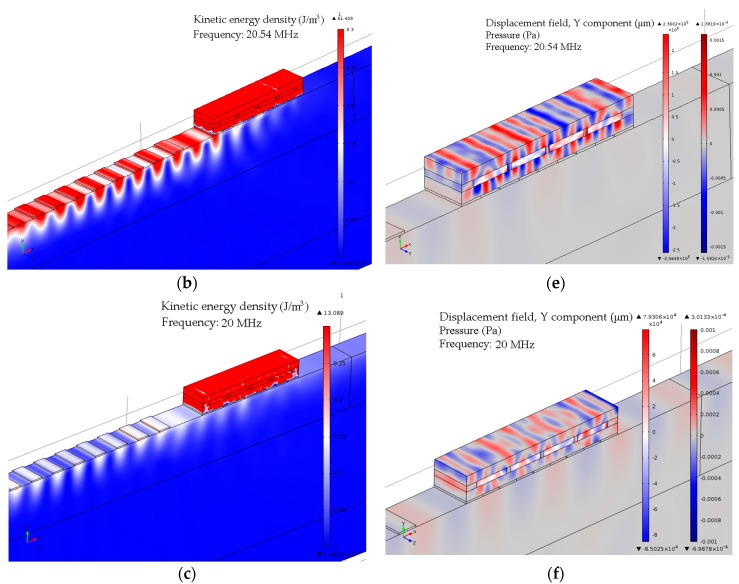
Kinetic energy density distribution at the SAW excitation frequency of (**a**) 20.45 MHz; (**b**) 20.54 MHz; (**c**) 20 MHz; displacement pattern and pressure distribution for the structure filled with liquid (speed of sound 1500 m/s) at the frequency of (**d**) 20.45 MHz; (**e**) 20.54 MHz; and (**f**) 20 MHz.

The simulation results demonstrate the existence of two transmission minima that are dependent on the speed of sound of the liquid confined in the structure channels. The analysis of the microfluidic structure resonance modes for the case when it is filled with the fluid (speed of sound 1500 m/s) is shown in Figure 4. The structure kinetic energy distribution and displacement field distribution are shown for the resonance frequencies (20.45 MHz and 20.54 MHz) and for the central frequency of SAW. As can be seen in Figure 4a,b, at the structure resonance frequency the acoustic energy of the incident surface wave is concentrated within the composite microfluidic arrangement and partially reflected back to the source. The displacement field distribution taken at the resonance frequencies shows a complex structure vibration pattern that is affected by the finite structure dimensions and confined within the arrangement liquid, Figure 4d,e. Structure behavior at the frequency different from resonances is demonstrated in Figure 4c,f. It can be seen that the structure reflects considerably less acoustic energy of incident surface wave that propagates under the structure with some attenuation. The displacement field distribution shows that the displacement magnitude of the microfluidic arrangement in the current case is comparable with the waveguide surface disturbance. 

The developed and fabricated sensor arrangement was experimentally investigated. Speed of sound is one of the informative parameters that reflects the liquid compressibility and, as a result, gains an access to the sensing properties that are directly influenced by the intermolecular interactions of liquid mixtures. It indicates possible deviations caused by non-linear mixture behavior. One vivid example is the mixtures of deionized (DI) water and 1-propanol. Despite the fact that 1-propanol has a lower speed of sound than DI water, the binary mixture of the two liquids results in an increase in the speed of sound for the molar fraction up to 0.058. This phenomenon was demonstrated in Reference [26]. It was shown that by adding alcohol to water, the properties of the mixture are affected in two ways. Firstly, the alcohol acts as one of the mixture components, contributing to its physical and chemical properties. Secondly, the presence of alcohol modifies the molecular structure of the water, changing the intermolecular interactions in the solution [26]. Similar to the detection of water-alcohol binary mixtures, the sensor can be utilized for a broad variety of applications, for example the detection of interactions within bio-substances, such as protein—protein, protein—DNA, antigen—antibody, and many other interactions. However, the scope of this work does not cover the experimental verification of the sensor application for certain bio-substance analyses, but instead demonstrates the ability of the novel microfluidic sensor platform.

**Figure 5:**
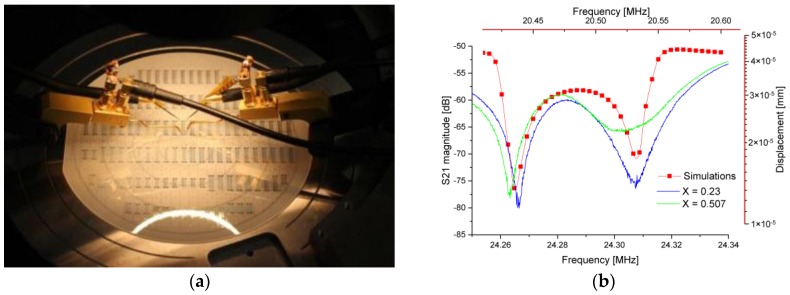
(**a**) Wafer scale measurement setup for the conduction of the sensor experimental investigations; (**b**) Theoretically simulated structure response taken as a displacement magnitude (red marked curve, red marked axis) for deionized (DI) water (speed of sound 1500 m/s) and measured sensor response for the liquid mixtures of DI water and 1-propanol of molar fraction X = 0.23 (blue curve, bottom X-axis) and X = 0.507 (green curve, bottom X-axis).

The sensor experimental verification was completed by utilizing a standard approach for SAW devices by the measurement of S-parameters. The sensor was designed as a two-port SAW device. Measurements of S_21_-parameters were performed without additional matching circuits. Agilent 4395A Network/Spectrum/Impedance analyzer with an S-parameter test set Agilent 87511A were utilized for the measurements. The measurements were conducted in the network analyzer mode of the instrument. A number of measuring points was set to the maximal limit of 801 points. The frequency sweep type was defined as linear. An intermediate frequency (IF) bandwidth was set to 1 kHz and a source power was set to be equal to 15 dBm. Manufactured sensors were measured directly on a wafer without single sensor dicing and packaging. The probe station with 73APT-100K kelvin probes was utilized for the experimental verifications. Analyzed liquids were manually pumped to the structure with the help of a micropipette. The experiments were conducted in an air conditioned laboratory, where the temperature was kept at 22 °C with possible deviations of 1 °C. The experimental data were obtained at stable and controlled temperature conditions within the same day. The experimental setup is given in Figure 5a. The measured S_21_-parameter sensor response in conjunction with the structure simulation results for DI water (1500 m/s) are shown in Figure 5b.

**Figure 6:**
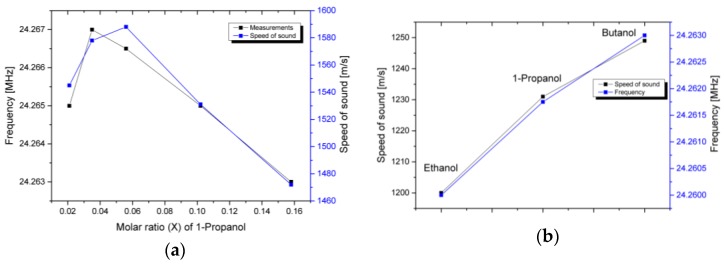
(**a**) Dependence of the transmission minima on the speed of sound of a binary mixture of water and 1-propanol for a molar fraction range of 0.021–0.158 [27]; (**b**) Dependence of the transmission minima on the speed of sound of standard alcohols (ethanol, 1-propanol, butanol) [27].

The experimentally measured curves are demonstrated for the mixture of DI water and 1-propanol for the molar fraction (X) of the linear dependence of the speed of sound versus composition. As can be seen, the increase of the 1-propanol content causes a shift of the measured dependences to the lower frequency range that correlates with the speed of sound variation. The lower frequency structure resonance shifts gradually almost without change in the quality factor when the higher frequency resonance deviates, depending on the liquid. Such a change in the quality factor can be explained by the rather complex vibration mechanism of the complete microfluidic arrangement excited with external surface waves. For sensing purposes, it is beneficial to control the position of the transmission dip that is not variable in terms of bandwidth. Therefore, the measurements of various mixtures were completed with the main focus on the position of the low frequency resonance mode. Experimental investigations of the structure response for mixtures of 1-propanol and DI water with a molar fraction in the range of 0.021–0.158 together with the speed of sound data [27] are shown in Figure 6a. As can be seen, the experimental curve and the data for the speed of sound of the mixture of deionized water and 1-propanol have a sufficiently high agreement. An initial increase of the 1-propanol concentration shifts the transmission minimum to the higher frequency. The further increase of the 1-propanol concentration turns the direction of the transmission minimum and shift to the lower frequency. The observable mismatch at low alcohol concentrations can be advocated by significant variation of the analyte surface tension that can affect the channel filling uniformity and result in measurement error. Similar experiments with the use of standard alcohols were performed to separate the speed of sound dependence from other factors. The summarized results of the measured structure response versus the speed of sound data are displayed in Figure 6b. The experimental curve corresponds with sufficient agreement to the speed of sound variation for the chosen alcohols.

The observable relation between the frequency of the transmission dip and the speed of sound of the fluid is rather complex. It depends on the resonant properties of the complete composite arrangement that consists of SU-8 polymer and the liquid analyte. It has been revealed that the excited structure modes correspond to the arrangement eigenmodes. However, the structure containment at the IDT boundaries and the liquid involvement in the structure resonances makes the analytical description of observed dependences very important. The involvement of the substrate with the structure resonance is supposed to be rather low because it is separated from the structure with electrodes, as experimentally observed. On the other hand, due to technological reasons, the fabricated arrangement can have less defined boundaries between the waveguide surface and the structure.

## 4. Discussion

In comparison to the SAW sensor concepts based on near-surface detection, the described sensor concept follows the idea of liquid confinement within the microfluidic structure. This is a distinguishing feature in comparison to existing microacoustic sensor approaches. The sensor principle behind this approach is the excitation of resonance modes in the solid-liquid composite arrangement that responds to properties of the liquid constituent. 

Referring to previously developed sensor structures [17,28,29,30], initial attempts were focused in a direction similar to previously developed structures, where the incident wave passes through the liquid-containing cylindrical inclusions that are periodically distributed along the propagation path. Unfortunately, this straightforward approach suffers from significant scattering of the incident surface wave into the substrate volume, resulting in considerable losses of the sensor signal. In addition, etching the structure within piezoelectric waveguides is rather complex in terms of the fulfilment of the required accuracy in all three dimensions [31,32]. As a result, the application of the sensor design that is similar to previously investigated millimeter-scale structures was insufficient for the SAW-based sensor arrangement. 

The SAW-based phononic sensor approach requires, on the one hand, the mode conversion of the SAW into the prevailing longitudinal mode in the liquid cavity. In the second step, this longitudinal mode must couple back to the surface wave without significant deterioration of the wavefront while passing through the periodic arrangement of liquid inclusions. This resynchronization with the SAW wavelength can make the detection of liquid cavity resonances rather complicated and inefficient. That leads to the necessity of introducing a coupling layer. The number of free design parameters increases dramatically, and a large set of different kinds of structural modes can be excited, including those where the liquid is involved. For the sensing purposes, it is advantageous to control the position of the transmission maxima (or minima) in contrast to the detection of the variation of the bandgap. Infinite periodic structures are subjected to exhibit a bandgap for a certain wavelength region, and the appearance of localized resonances in those structures is possible to achieve only with symmetry disruption. Finite structures, on the other hand, are able to exhibit the structural resonances in rather uniform periodic arrangements. In this case, the complete regular periodic arrangement within its finite dimensions with prescribed boundaries supports the resonance structural modes at certain wavelengths. The behavior of the finite composite arrangements was previously analytically described in Reference [33]. It was shown that for the two-dimensional arrangements as well as for a one-dimensional case, the free boundaries cause local resonances that affect the response within the bandgap. Another work showed that the local resonances in a structure constituent contribute in arrangement response. In Reference [34], the study of periodical pillar arrangement integrated into a SAW device revealed that low frequency bandgap arises because of local resonances in the scatters. At the wavelength of localized resonance, the acoustic energy of the incident surface wave is strongly confined in structure constituents. Design optimization becomes two-fold: the realization of highly localized structure resonances that can be utilized for liquid sensing purposes, and the efficient coupling of a surface wave to the periodic arrangement. On the other hand, the phononic structure that performs these tasks must suppress any unwanted scattering or propagation of any acoustic wave into the bulk of the piezoelectric substrate. An excitation of any kind of the structure resonance can cause immediate wave scattering into the bulk. To avoid that, the periodic over-layer structure has to isolate the excited resonance from the piezoelectric carrier. The solution was found by moving the over-layer structure above the receiving IDTs and analyzing the transmission minima that correspond to the modes of a high acoustic energy concentration within the over-layer structure. The operating scheme can be described by the following sequence. The over-layer structure couples the acoustic energy from the incident surface acoustic wave only at specific frequencies that correspond to structural resonance modes. Among all possible structure modes, the most relevant have a liquid involvement. The induced structure resonances are isolated by the periodic arrangement from the waveguide surface.

The experimentally obtained structure response is shifted in the frequency in comparison to experimental observations, which apparently is a consequence of technological tolerances with the fabricated structure, as well as possible deviation between model-defined material properties and the fabricated sensor. Although the role of the periodic structure in this particular case is different from commonly applied schemes, we believe that it allows for the most efficient implementation of the phononic crystal concept into the SAW platform. The periodic arrangement of microfluidic channels operates as a composite solid-liquid structure that couples the propagation along the waveguide surface wave and concentrates the acoustic energy within the structure at frequencies of structural resonances. The frequency of excited resonances within the structure is dependent on the material properties of each of the constituents. The variation of material parameters of one of the constituents causes the shift of the structural resonance that is detected as a frequency transmission minimum.

## 5. Conclusions

In this work, the novel liquid sensor concept that utilizes the control of structural resonances in a system of periodically placed microfluidic arrangement was introduced. The concept merges advantages of the SAW sensor platform and ultrasonic velocimetry methods. It allows for the utilization of the conventional SAW arrangement and the detection of properties of the liquid that is trapped within the structure. The paper introduced the approach that allows for the detection of the volumetric properties of a liquid analyte, such as the speed of sound, with a SAW sensor similar to ultrasonic velocimetry methods. The developed SAW-based sensor completes the sensor within the planar technological approach and makes it compatible with Lab-on-a-Chip platforms. The obvious necessity for further structure improvement with respect to the fabrication technology and systematic analysis of other possible arrangements should be mentioned, and will be the subject of upcoming research. The demonstrated theoretical and experimental results described in the current contribution prove the feasibility of the proposed sensor concept.
